# Endocannabinoid and *N*-acylethanolamine concentrations in hair of female patients with posttraumatic stress disorder – associations with clinical symptoms and outcomes following multimodal trauma-focused inpatient treatment

**DOI:** 10.1038/s41398-025-03476-3

**Published:** 2025-08-23

**Authors:** L. Bergunde, M. L. Woud, L. Shkreli, L. Schindler-Gmelch, S. Garthus-Niegel, S. E. Blackwell, C. Kirschbaum, H. Kessler, S. Steudte-Schmiedgen

**Affiliations:** 1 Institute and Policlinic of Occupational and Social Medicine, Faculty of Medicine and University Hospital Carl Gustav Carus, TUD Dresden University of Technology, Dresden, Germany; 2 Department of Psychotherapy and Psychosomatic Medicine, Faculty of Medicine and University Hospital Carl Gustav Carus, TUD Dresden University of Technology, Dresden, Germany; 3 Department of Clinical Psychology and Experimental Psychopathology, Georg-Elias-Mueller-Institute of Psychology, Georg-August-Universität Göttingen, Göttingen, Germany; 4 Mental Health Research and Treatment Center, Ruhr-Universität Bochum, Bochum, Germany; 5 Department of Psychiatry, University of Oxford, Oxford, UK; 6 Department of Clinical Psychology and Psychotherapy, Friedrich-Alexander-Universität Erlangen-Nürnberg, Erlangen, Germany; 7 Institute for Systems Medicine (ISM), Faculty of Medicine, Medical School Hamburg MSH, Hamburg, Germany; 8 Department of Childhood and Families, Norwegian Institute of Public Health, Oslo, Norway; 9 Institute of Biological Psychology, Faculty of Psychology, Technische Universität Dresden, Dresden, Germany; 10 Department of Psychosomatic Medicine and Psychotherapy, Campus Fulda, University of Marburg, Fulda, Germany; 11 Department of Psychosomatic Medicine and Psychotherapy, LWL-University Hospital, Ruhr-University Bochum, Bochum, Germany

**Keywords:** Prognostic markers, Human behaviour

## Abstract

While psychotherapeutic treatments for posttraumatic stress disorder (PTSD) show in general good responses in affected individuals, 30–40% of patients show limited improvement. On a biological level, the endocannabinoid system of the body may play a role in the aftermath of trauma, in PTSD, and in extinction processes. This study is a secondary analysis of a randomized-controlled trial including patients with PTSD over the course of trauma-focused inpatient treatment. It aimed to investigate whether endocannabinoid system alterations are associated with symptom severity and treatment response. Fifty-four female inpatients with PTSD provided hair samples and completed psychometric questionnaires at pre-treatment, post-treatment, and 3-month follow-up. Endocannabinoid (EC: AEA, 1-AG/2-AG) and *N*-acylethanolamine (NAE: SEA, PEA, OEA) concentrations were measured in scalp-near 3-cm hair segments, reflecting cumulative concentrations in the 3 months prior to sampling. At pre-treatment, higher depressive and anxiety symptoms were significantly associated with lower hair AEA levels, whereas higher PTSD symptoms (when controlling for depressive symptoms) and more traumatic experiences were significantly associated with higher hair AEA and NAE levels respectively. PTSD symptoms improved across treatment, remaining stable at 3-month follow-up, but were predicted neither by pre-treatment hair ECs/NAEs nor their changes across treatment and follow-up, which was confirmed in subgroup analyses. Our findings suggest that hair ECs/NAEs may be distinctly linked with trauma-related and affective and anxiety symptoms, however, do not predict treatment response in PTSD. This challenges expectations and highlights the complexity of endocannabinoid system alterations in stress-related psychopathology. Given the study’s limitations, including a female-only sample and lack of a control group, larger studies with control groups and multiple biomarkers are needed to identify intervention-related biomarkers in PTSD.

## Highlights

- Hair endocannabinoids and *N*-acylethanolamines at pre-treatment and their change were unrelated to PTSD symptoms across treatment and follow-up
- At pre-treatment, hair AEA associated negatively with pre-treatment depressive and anxiety symptoms
- At pre-treatment, hair AEA associated positively with PTSD symptoms after controlling for depressive symptoms
- At pre-treatment, more traumatic experiences were related to higher hair SEA, PEA, and OEA levels in female inpatients with PTSD

## Introduction

Posttraumatic stress disorder (PTSD) is a persistent mental health condition with a 3.9% cross-national lifetime prevalence [1], and is associated with high subjective and economic burdens [2]. PTSD has been characterized as a maladaptive response to trauma exposure that occurs in a subset of trauma-exposed individuals, involving maladaptive memory consolidation, deficits in fear extinction, and a failed reinstatement of physiological homeostasis [3, 4]. Meta-analytic findings confirm large effect sizes of trauma-focused exposure therapy for PTSD [5], and these treatments ameliorate extinction recall deficits [6]. However, meta-analyses also suggest that only between 54.8% – 63.5% of patients with PTSD remit to the extent that they no longer meet the diagnostic criteria at follow-up [7, 8]. Consequently, research aims to identify biological markers in relation to PTSD to inform prevention, identification, and treatment effectiveness [9]. These include 1) susceptibility biomarkers, being assessed before or after exposure to traumatic events and indicative of the risk of developing PTSD, 2) diagnostic biomarkers, which are measured after traumatic experiences and identify individuals with PTSD, 3) resilience biomarkers, which are also measured post-trauma and identify individuals without PTSD symptom progression, and finally 4) intervention-related biomarkers, which are assessed before and throughout treatment and allow prediction and monitoring of treatment response, with the goal of optimizing treatment matching in terms of personalized medicine [9–11]. In this context, components of the endocannabinoid system (ECS) have been increasingly studied, both as a novel biomarker candidate and possible treatment target for PTSD [12, 13].

The ECS is a widespread lipid signaling system including the ligands *N*-arachidonoylethanolamine (AEA; anandamide) and 2-arachidonoylglycerol (2-AG). These are retrograde messengers synthesized “on demand” that bind to pre-synaptic G protein-coupled receptors (cannabinoid receptors CB1 and CB2), thereby modulating various neurotransmitter systems (e.g., GABA, glutamate, dopamine). It further encompasses enzymes that metabolize and degrade AEA (fatty acid amide hydrolase (FAAH)) and 2-AG (monoacylglycerol lipase (MAGL)), as well as the *N*-acylethanolamines (NAEs) SEA, PEA and OEA [14]. The latter exert so-called ‘entourage’ effects potentiating the effects of AEA [15] by binding to other receptors in brain and periphery, producing amongst others anti-inflammatory, neuroprotective, and analgesic effects (reviewed in [16]). The ECS is involved in many physiological homeostatic processes relating to pain, feeding, inflammation [17, 18] thermoregulation [19], sleep [20], plays a role in (emotional) memory [12, 21] and fear extinction [12, 22], and has been shown to interact with biological stress systems, in particular the hypothalamic-pituitary-adrenal axis including the end product cortisol [23]. Several of these processes are relevant to PTSD pathogenesis and treatment [12], and preclinical and human research indicates that alterations in ECS components and signaling play a role in stress- and trauma-related disorders [13]. Emerging evidence indicates an association between reduced ECS signaling and PTSD [24, 25], depressive disorders [26], and anxiety symptoms [27]. Preclinical studies suggest that augmenting AEA and 2-AG levels can reduce anxiety- and depression-like behaviors following acute and chronic stress [28–32], while translational research with humans, for instance via genetic polymorphisms in the human FAAH gene or administration of FAAH inhibitors, shows that increased AEA levels can confer anxiolytic and stress-resilient effects [33, 34]. Nevertheless, the direction and causal relationship between PTSD and ECS alterations remains unclear, warranting further research to determine whether these alterations reflect a vulnerability factor to PTSD or result from trauma exposure and/or PTSD symptomatology, and whether potential changes ameliorate with or influence treatment response [12, 25].

Regarding PTSD pathophysiology, increasing evidence implicates changes in the ECS after traumatic stress as well as to the development of PTSD [12, 23]. In rodent models, for example, exposure to early, chronic, and later life stress was associated with alterations in CB receptor expression and tissue EC content in the short- and long-term [35]. In humans, exposure to a greater number of traumatic events has been linked to greater risk of PTSD [36], greater symptom severity [37], and lower likelihood of spontaneous remission [38]. These dose-response relationships have been termed the “building-block effect” of traumatic experiences, in which each additional traumatic event increases PTSD risk, potentially via changes to long-term reduced basal cortisol secretion [39], and possibly altered EC/NAE output and stress reactivity [23, 40, 41]. Cross-sectional research with humans has indicated an association of PTSD risk and severity with alterations in basal circulating blood ECs. However, results have been inconsistent, with studies indicating downregulated EC concentrations [24, 42–44], while others [45–48], including a recent meta-analysis [49], point towards an upregulation, and others found no evidence of EC-related changes in individuals with PTSD compared to healthy controls (e.g., [50]). Similarly, findings linking PTSD symptom severity to circulating EC/NAE levels have been inconsistent and varied by symptom sub-scale (negative [24, 42]; positive [46, 50]). Inconsistencies may stem from small, mixed-sex samples, despite evidence of ECS sex differences [51, 52], varied index traumas (e.g., war, injury, intimate partner violence), and limited consideration of genetic polymorphisms like *rs324420* in FAAH [48] and *rs806371* in CNR1 [45] which have been associated with PTSD severity. Additionally, dynamic blood EC concentrations influenced by situational factors may further contribute to the observed heterogeneity [53].

Recently, EC/NAE levels have also been quantified in hair and are assumed to reflect longer-term EC/NAE secretion over weeks to months less affected by acute situational influences [54, 55]. Initial research employing hair ECs/NAEs found reduced hair OEA levels in rebel war survivors with versus without PTSD as well as negative associations of hair NAE levels with number of traumatic events and PTSD symptom severity, respectively [41]. In community samples, hair EC/NAE levels were also negatively associated with burnout and anxiety symptoms (AEA; [54]), childbirth-related PTSD symptoms (AEA; [56]), and depressive symptoms (AEA; [57]) and reduced in health-care workers with anxiety (SEA and PEA; [58]). This is in line with preclinical evidence and the idea that a hypoactive ECS may increase vulnerability in stress-related psychopathology [12]. However, some studies found positive or no associations between hair ECs/NAEs and trauma and PTSD, or depressive, and anxiety symptoms (e.g., [59–61]), highlighting the need for further clarification.

Regarding PTSD treatment, trauma-focused therapies often include elements of (imaginative) exposure, and are conceptually based on the mechanisms of fear conditioning and extinction learning [62]. The ECS plays a prominent role in fear extinction and extinction memory [33] with ECs maintaining a balance between activity of excitatory and inhibitory neurons in the amygdala [63] and CB1 receptors being highly expressed in areas of the limbic system (including the prefrontal cortex, amygdala, and hippocampus) relevant to fear extinction learning [22]. A recent fMRI study found that the degree of neural activation during extinction learning correlated positively with blood AEA levels [64] and translational evidence by Mayo et al. [65] found that healthy individuals with a genetically less active FAAH enzyme had elevated basal AEA levels, better fear extinction, and extinction recall. Taken together, these findings support ECS alterations in the aftermath of traumatic stress and that poor treatment response in PTSD could be attributable to deficits in extinction learning related to altered ECS functioning, particularly in relation to AEA [25, 33].

Thus, research examining ECs/NAEs in relation to PTSD seems promising to augment treatment outcomes. While several studies have examined endocrine, genetic, and neural predictors of PTSD treatment efficacy [10, 66], to our knowledge only one study has examined predictive effects of EC levels for treatment outcome specifically, showing no predictive effect of pre-treatment AEA or 2-AG blood plasma concentrations in male war veterans diagnosed with PTSD [50]. However, based on available evidence highlighting elevated AEA as beneficial for fear extinction learning and recall [64, 65], it seems plausible that elevated ECs/NAEs at pre-treatment and increases in ECs/NAEs across treatment and follow-up may be associated with better treatment outcomes [63, 64]. Therefore, investigations with a non-military, inpatient population examining effects both immediately following treatment as well as at follow-up are outstanding and would benefit from including assessment of *hair* ECs/NAEs to reflect more long-term ECS functioning.

The present study is a secondary analysis on data from a randomized-controlled trial (RCT) investigating the effects of a computerized training intervention, cognitive bias modification for appraisals (CBM-APP), in inpatients with PTSD [67]. Previous analyses revealed that patients receiving CBM-APP versus sham training showed greater reductions in clinical outcome measures (i.e., posttraumatic cognitions, dysfunctional appraisals, and PTSD symptoms) post-treatment [68]. A re-analysis examining the role of hair steroids (i.e., hair cortisol, cortisone, and DHEA) found no robust cross-sectional or predictive associations with PTSD symptoms across treatment [69]. Thus, this re-analysis had two aims: First, baseline associations between pre-treatment hair ECs/NAEs and pre-treatment lifetime trauma exposure, PTSD, depressive, and anxiety symptom severity were investigated, hypothesizing negative relationships based on prior human [12, 26, 27] and pre-clinical [70] evidence. Secondly, we examined whether hair ECs/NAEs predicted PTSD treatment response [64]. Specifically, we tested if (i) higher pre-treatment hair ECs/NAEs, and (ii) increases in hair EC/NAE levels across treatment and follow-up predicted greater PTSD symptom improvement over time. Finally, given the ECS’s role in extinction learning, a key mechanism of exposure therapy [62], we explored these effects in a subsample of patients who received exposure during treatment.

## Methods

### Participants and procedure

**Fig. 1:**
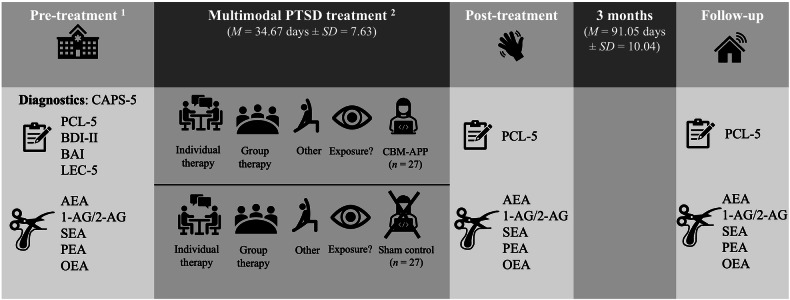
Assessment points from pre-treatment to 3-month follow-up. *Notes*. CAPS-5 Clinician Administered PTSD Scale for DSM-5. PCL-5  PTSD Checklist for DSM-5. BDI-II Beck Depression Inventory. BAI Beck Anxiety Inventory. LEC-5 Life Events Checklist for DSM-5. CBM-APP Cognitive Bias Modification for Appraisal Training. ^1^ While the RCT included additional assessments, the present investigation focused on assessments where hair samples were taken: (1) At pre-treatment, PTSD diagnosis was confirmed, baseline questionnaires assessing current symptomatology were completed, and a hair sample was provided (*n* = 53). (2) At post-treatment (*n* = 53) and at the (3) 3-month follow-up (*n* = 38), patients also completed PTSD symptom questionnaires and provided hair samples. This resulted in a total of *n* = 54 patients’ data being included. ^2^ The clinic offers multimodal inpatient treatment for PTSD typically lasting eight weeks, with a typical week including one session of individual CBT therapy, group therapy, and other therapies (e.g., physiotherapy). Some patients received exposure-based treatments and due to the RCT, half of the patients received either CBM-APP or a sham control training during the first treatment phase.

Participants were a subsample of a RCT (trial registration number NCT02687555) investigating the effects of CBM-APP in inpatients with PTSD (*N* = 80) recruited at the Department of Psychosomatic Medicine and Psychotherapy, LWL-University Clinic of Ruhr-Universität Bochum, Germany (for details, see [67]). Patients underwent an 8-week standard inpatient treatment program for PTSD, which was based on Cognitive Behavioural Therapy (CBT) and involved a multimodal approach. Each week typically included one individual CBT session, three trauma-focused group therapy sessions, two trauma stabilization group sessions, two kinesiotherapy sessions, two art therapy sessions, physiotherapy, clinical rounds, and daily brief check-ins with a nurse. Exposure-based treatments were conducted with some but not all patients due to some patients not meeting the requirements for exposure therapy yet. For these cases, the focus was on stabilization and preparation for trauma processing. Also, due to the RCT, during the first treatment phase, participants were randomly assigned to receive either CBM-APP or a sham control training (Fig. 1). The study was approved by the Faculty of Medicine, Ruhr-Universität Bochum ethics committee (15-5477) and informed consent according to the Declaration of Helsinki was obtained. Research adhered to the guidelines for Good Scientific Practice and the laboratory, while not subject to the guidelines for Good Laboratory Practice, consistently works in accordance with all relevant specifications of laboratory safety and reliability.

RCT eligibility criteria were being aged 18–60 years, being fluent in German, and having a primary PTSD diagnosis according to ICD-10 (F43.1; [71]) and DSM-5 [72], confirmed at pre-treatment with the Clinician-Administered PTSD Scale for DSM-5 (CAPS-5; [73]). Participants were excluded if they reported active suicidality, substance abuse/dependence currently or during the past six months, a past or present psychotic disorder, learning disability/intellectual impairment, and red/green colour blindness or non-correct vision impairment (due to the RCT). To be considered for the present investigation, participants had to have provided hair samples with 3 cm for analysis at least once at pre-treatment, post-treatment, or follow-up (*N* = 67). As only *n* = 2 of these 67 patients were male, these were excluded due to research indicating ECS sex differences [51, 52]. Furthermore, *n* = 8 individuals reporting an intake of glucocorticoid-containing or hormonal medications (excluding oral contraceptives) and *n* = 3 with a diagnosis of diabetes were excluded. This was implemented as these factors may directly affect the ECS [74] or the glucocorticoid system [75–77], which through close coupling in turn may indirectly impact the ECS [78]. This resulted in available data of *n* = 54, (*n* = 53 at pre-treatment, *n* = 53 at post-treatment and *n* = 38 at 3-month follow-up; see Fig. 1), with *n* = 16 (29.6%) no longer participating at follow-up. Attrition analyses were calculated using Welch’s t-test and Chi-square test, showing that completers and non-completers did not differ significantly regarding sociodemographic, clinical, or hair EC/NAE data except for non-completers having lower hair SEA concentrations at pre-treatment (*t*(40.87) = 2.25, *p* = .030; see supplements Tables S1 and S2 for full attrition analyses).

Descriptive characteristics of the study sample of 54 female inpatients can be found in Table 1. Average age was 40 years (*SD* = 12.29) and average number of ICD-10 F-diagnoses including PTSD at pre-treatment was 2.61. Participants had experienced on average 7.35 (*SD* = 2.93) potentially traumatic events reported in the Life Events Checklist (LEC-5). In addition to PTSD, the most frequent clinical diagnoses were affective disorders. Severe anxiety (BAI ≥ 26) and depressive (BDI-II ≥ 29) symptoms were reported by 74.1% and 81.5% of patients, respectively. Except for two, all patients reported medication intake, and average BMI was within the obesity range. The hospital stay lasted on average 6.8 weeks (*SD* = 1.35) and 68.5% of included patients received exposure therapy during inpatient treatment.

**Table 1:** Sociodemographic, clinical, and hair-related characteristics (*N* = 54).

Sample characteristics	*n* (%) or Mean ± *SD* (range)
**Sociodemographic variables**	
Age in years	40.11 ± 12.29 (19 – 58)
Body Mass Index (BMI)	31.16 ± 9.51 (18.93 – 63.75)
CBM-APP Group	27 (50.0)
Married	18 (33.3)
Migration background	9 (16.7)
Smoking	26 (48.1)
Heavy smoking ≥15 cigarettes/day	15 (27.8)
**Medical variables**	
Length of hospital stay in days	47.65 ± 9.42 (15 – 65)
Received exposure therapy	37 (68.5)
Number of F diagnoses ^a^	2.61 ± 0.76 (2 – 5)
*Psychological comorbidities*	
Any	54 (100)
Major depressive disorder	52 (96.3)
Eating disorders	8 (14.8)
Chronic pain disorder	7 (13.0)
Borderline personality disorder	6 (11.1)
Anxiety disorder	4 (7.4)
Substance dependence/abuse ^b^	3 (5.6)
Other (e.g., ADHD)	5 (9.3)
*Somatic comorbidities*	
Patients with comorbidities total	19 (35.2)
Hypothyroidism	11 (20.4)
Hypertension	7 (13.0)
Asthma	3 (5.6)
Other (e.g., chronic gastritis)	8 (14.8)
*Medication intake*	
Any	52 (96.30)
Changes in medication during treatment	36 (66.7)
Patients taking psychotropic medication	47 (87.0)
Antidepressants ^c^	40 (74.07)
Quetiapine	11 (20.37)
Promethazine	6 (11.11)
Pipamperone	3 (5.56)
Valproic acid	3 (5.56)
Opioids	3 (5.56)
Lamotrigine	3 (5.56)
Pregabalin	3 (5.56)
Other	16 (29.63)
Non-psychotropic medication intake ^d^	32 (59.3)
**Clinical-psychological variables at pre-treatment**	
Depressive symptoms, BDI-II	35.74 ± 8.45 (16 – 52)
Anxiety symptoms, BAI	31.28 ± 10.91 (4 – 55)
CAPS total score	43.26 ± 8.77 (26 – 71)
LEC-5 score	7.35 ± 2.93 (2 – 15)
Weighted LEC-5 score	18.57 ± 7.23 (6 – 35)
Index Trauma	
Sexual Violence	32 (59.26)
Physical Violence	11 (20.37)
Severe Accident	4 (7.41)
Other	7 (12.96)
Years since worst traumatic experience	22.32 ± 16.31 (0.25 – 51)

*CBM-APP* cognitive bias modification for appraisal, *BDI-II* beck depression inventory I, *BAI* beck anxiety inventory, *CAPS* clinician administered PTSD scale, *LEC-5* life events checklist for DSM-5, *ADHD* attention deficit hyperactivity disorder.

^a^including the primary clinical diagnosis.

^b^diagnosis prior to six months before admission.

^c^antidepressants include selective serotonin reuptake inhibitors (SSRIs), monoamine oxidase inhibitors (MAOIs), tricyclic antidepressants and atypical antidepressants.

^d^including thyroid medication, beta blockers and others.

### Measures

#### Psychological measures

Information on **sociodemographic** (e.g., age) and **clinical variables** (e.g., medication intake) were taken from clinical records and **hair-related variables** (e.g., hair mass, storage time) were obtained from laboratory documentation.

**Lifetime trauma exposure** was measured using the Life Events Checklist for DSM-5 (LEC-5; [79]). The LEC-5 asks about experiences of 16 potentially traumatic events (e.g., sexual assault, traffic accidents) as well as about other stressful experiences not listed, resulting in a maximum score of 17. Participants can choose one of six levels of exposure: “happened to me”, “witnessed it”, “learned about it”, “part of my job”, “not sure”, and “doesn’t apply”. We calculated a weighted total score assigning weights 3 (“happened to me”), 2 (“witnessed it”), 1 (“learned about it”) and 0 (all other) [80].

**PTSD symptoms** as the primary outcome variable for these analyses were assessed with the German version of the PTSD Checklist for DSM-5 (PCL-5; [81]) at all assessment points. The 20-item questionnaire assesses PTSD symptoms according to DSM-5 (four symptom clusters: re-experiencing, avoidance, negative alterations in cognitions and mood, and hyperarousal). Participants rated how strongly they experienced each symptom during the previous week on a 5-point Likert scale ranging from 0 (not at all) to 4 (strongly). The total score showed acceptable to excellent internal reliability (Cronbach’s alpha = .73 – .96) and was used in the main analyses. For exploratory subscale analyses at pre-treatment the four subscales were included, demonstrating unacceptable to questionable internal reliability at pre-treatment (re-experiencing (.69), avoidance (.41), negative cognitions and mood (.20), hyperarousal (.61)).

**Depressive symptoms** at pre-treatment were assessed with the German version of the Beck Depression Inventory-II (BDI-II; [82]). The BDI-II is a widely used self-report questionnaire consisting of 21 items assessing depressive symptoms over the previous two weeks using a 4-point Likert scale and showed good internal reliability (Cronbach’s alpha = .84). For exploratory analyses at pre-treatment, we also examined the three subscales cognitive, affective, and somatic symptoms, suggested by research [83], which demonstrated questionable to acceptable internal reliability (Cronbach’s alpha: .78 (cognitive), .67 (affective), .63 (somatic)).

**Anxiety symptoms** were assessed at pre-treatment using the German version of the Beck Anxiety Inventory (BAI; [84]). The BAI has evidenced good psychometric characteristics, and internal reliability was good in this study (Cronbach’s alpha = .87). Patients self-report the degree of anxiety symptoms over the past week on 21 items using a 4-point Likert scale.

#### Long-term EC/NAE output

Hair samples were taken at pre-treatment, at post-treatment, and 3-month follow-up (see Fig. 1) and sent to the biochemical laboratory at Technische Universität Dresden (Prof. Kirschbaum). In scalp-near 3 cm hair segments, concentrations of ECs/NAEs (AEA, 1-AG/2-AG, SEA, PEA, OEA) were quantified using an established protocol for liquid chromatography tandem mass spectrometry (LC-MS/MS) [54] (for details, please see supplementary materials Information S1). During extraction, 2-AG isomerizes easily to 1-arachidonoyl-sn-glycerol (1-AG) due to acyl group migration [85], complicating precise determination of 2-AG concentrations. Hence, as 1-AG is assumed to originate primarily from 2-AG, 2-AG and 1-AG levels were summed as one variable [54, 86]. As hair grows at a rate of about 1 cm per month [87], concentrations are assumed to reflect the 3 months of EC/NAE secretion prior to sampling.

### Data preparation and statistical analyses

SPSS version 29 and R version 4.4.2 [88] were used. Statistical significance was assessed using two-sided tests at a 5% significance level and with confidence intervals excluding zero indicating significance. As hair EC/NAE data were not normally distributed, log-transformations were applied, reducing skewness and improving normality. A fixed-value imputation-based approach was applied for (1) dealing with non-detectable values (only applicable for AEA: pre-treatment *n* = 4 (7.5%), post-treatment *n* = 7 (13.2%), and follow-up *n* = 2 (5.26%)) where these were replaced with the lowest measurable value for AEA (0.064 pg/mg) and (2) dealing with outliers ± 3 *SD*s (AEA: post-treatment *n* = 1) by replacing them with the next-highest value within 3 *SD*s [89, 90].

Preparatory analyses using Pearson correlations investigated associations between hair ECs/NAEs and showed that none of the variables identified from the literature that may influence hair ECs/NAEs (i.e., age, body mass index (BMI), hair mass, and storage time; [54, 56]) were consistently associated with hair ECs/NAEs in this study, hence these were not included as covariates (see supplements Table S3).

To investigate cross-sectional pre-treatment associations between hair ECs/NAEs, lifetime trauma exposure, and symptom severity of PTSD, depressive, and anxiety symptoms, Pearson correlations were calculated. To partial out potential confounding between depressive and PTSD symptoms, a partial correlational analysis was conducted between hair ECs/NAEs and PTSD symptoms, controlling for depressive symptoms and vice versa. All correlations were run with 2000 bootstrap samples to increase robustness. A post-hoc power analysis with G*power [91] showed that with *n* = 54 participants and an alpha-level of *p* = 0.05, this study had 11%, 61%, and 98% in correlational analyses, and 30%, 95%, and 100% power in approximated repeated-measures analyses to detect a small (*f* = 0.10), moderate (*f* = 0.25), and large effect (*f* = 0.40), respectively.

To examine the course of PTSD symptoms and hair ECs/NAEs across treatment and follow-up, we fitted linear mixed-effects models (LMMs) with a random intercept for participants and a fixed effect of time (pre-treatment, post-treatment, 3-month follow-up), group (CBM-APP yes/no), treatment duration, and lifetime trauma exposure for the outcomes PCL-5 and hair ECs/NAEs, respectively. To examine the predictive value of pre-treatment hair ECs/NAEs and change in hair ECs/NAEs on PCL-5 scores across treatment and follow-up, we fitted random intercept models predicting PCL-5 across time, adjusting for group (CBM-APP yes/no), treatment duration, and lifetime trauma exposure with (1) a fixed effect of pre-treatment hair ECs/NAEs (continuous variable) and the interaction of pre-treatment hair ECs/NAEs with time; and (2) change in hair ECs/NAEs (dichotomous variable with 0 = decrease in ECs/NAEs and 1 = increase in ECs/NAEs; once from pre-treatment to post-treatment and once from post-treatment to follow-up) and the interaction of hair EC/NAE change with time. The parameters of interest were the fixed interactions of pre-treatment hair ECs/NAEs and hair EC/NAE change with time respectively. Given the small sample size and some model assumptions (i.e., homoscedasticity of model residuals) not being met, we calculated robust LMMs using the *robustlmm* package [92] with maximum likelihood estimation. As robust LMMs do not provide overall model statistics, we performed post-hoc tests with the false discovery rate adjustment for multiple comparisons using the *emmeans* [93] package.

## Results

### Pre-treatment analyses

Pearson correlations showed that at each measurement occasion, AEA and 1-AG/2-AG (*rs* > 0.48), and SEA, PEA, and OEA (*rs* > 0.72) were significantly positively associated with one another, respectively (see supplements Table S4).

**Fig. 2:**
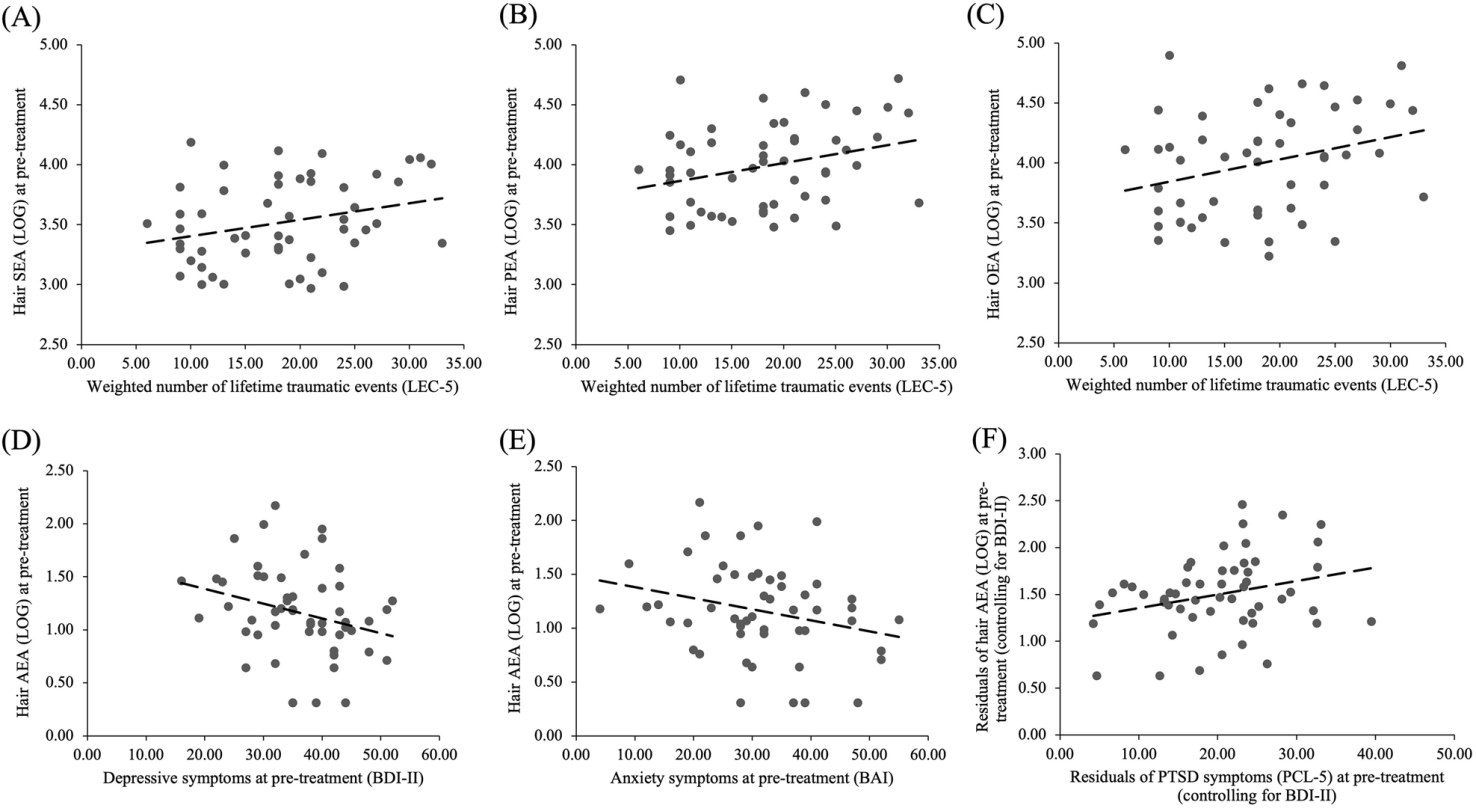
Scatterplots of pre-treatment associations between hair ECs/NAEs and weighted number of traumatic events and depressive, anxiety, and PTSD symptoms. *Note*. **A** Association between pre-treatment hair SEA and weighted number of traumatic events (*r* = .27, BCa 95% CI [.03, .51]). **B** Association between pre-treatment hair PEA and weighted number of traumatic events (*r* = .29, BCa 95% CI [.01, .53]). **C** Association between pre-treatment hair OEA and weighted number of traumatic events (*r* = .30, BCa 95% CI [.02, .54]). **D** Association between pre-treatment hair AEA and depressive symptoms (*r* = −.27, BCa 95% CI [−.46, −.07]). **E** Association between pre-treatment hair AEA and anxiety symptoms (*r* = −.26, BCa 95% CI [−.45, −.05]). **F** Association between pre-treatment hair AEA and PTSD symptoms, adjusted for BDI-II (*r* = .28, BCa 95% CI [.02, .55]). For visualisation the residuals of hair AEA and PTSD symptoms, controlling for BDI-II, were used in **F** and a constant of 1.5 was added to logarithmized hair AEA values for visualisation purposes in **D**−**G** and a constant of 20 was added to residuals of PTSD symptoms in **F** also for visualisation purposes only. A weighted score of lifetime traumatic events (LEC-5) was used with personally experienced events being weighted with 3, witnessed events with 2, and heard about events with 1. *BDI-II* Beck Depression Inventory. *BAI* Beck Anxiety Inventory. *PCL-5* PTSD Checklist for DSM-5. *LEC-5* Life Events Checklist for DSM-5.

With regard to clinical variables (see Table 2, Fig. 2), results showed a positive association between greater lifetime trauma exposure at pre-treatment and higher hair SEA, PEA and OEA levels. Associations with hair AEA and 1-AG/2-AG were not significant. Further, significant inverse associations between hair AEA and BDI-II, and hair AEA and BAI, but not hair AEA and PCL-5 scores were found. Exploratory subscale analyses (see supplementary materials Table S5) revealed that hair AEA was significantly negatively associated with the BDI-II affective (*r* = −.32, 95% BCa CI [−.53, −.11]) and cognitive subscales (*r* = −.36, 95% BCa CI [−.46, −.06]), whereas none of the PCL-5 subscales demonstrated significant associations with hair ECs/NAEs, albeit the hyperarousal subscale showing a non-significant trend with hair AEA (*r* = .24, 95% BCa CI [−.02, .48]). Partial correlation analyses between pre-treatment hair ECs/NAEs and PCL-5 scores, adjusting for BDI-II scores and vice versa, indicated a significant positive association between hair AEA and PCL-5 and confirmed the negative association between hair AEA and BDI-II. All other effects were not significant (Table 2).

**Table 2:** Pearson correlations between pre-treatment hair ECs/NAEs, lifetime trauma exposure, and pre-treatment symptom severity (*N* = 53).

	AEA T0			AG T0			SEA T0			PEA T0			OEA T0		
	*r*	BCa 95% CI	*p*	*r*	BCa 95% CI	*p*	*r*	BCa 95% CI	*p*	*r*	BCa 95% CI	*p*	*r*	BCa 95% CI	*p*
LEC-5	.15	−.14, .41	.312	.01	−.25, .27	.956	**.27**	**.03, .51**	**.035**	**.29**	**.01, .53**	**.039**	**.30**	**.02, .54**	**.030**
BDI-II T0	**−.27**	**−.46, −.07**	**.011**	−.15	−.47, .21	.357	.02	−.25, .26	.912	−.12	−.40, .15	.392	−.08	−.36, .21	.638
BAI T0	**−.26**	**−.45, −.05**	**.022**	−.17	−.44, .12	.213	−.01	−.27, .25	.930	−.19	−.46, .08	.178	−.22	−.49, .07	.159
PCL-5 T0	.12	−.13, .36	.351	.06	−.16, .27	.636	.03	−.23, .27	.821	−.07	−.34, .18	.575	−.05	−.30, .21	.741
*Controlled for PCL-5*															
BDI-II T0	**−.36**	**−.55, −.15**	**.008**	−.19	−.50, .13	.181	.01	−.29, .29	.969	−.10	−.36, .14	.484	−.06	−.37, .22	.655
*Controlled for BDI-II*															
PCL-5 T0	**.28**	**.02, .55**	**.047**	.13	−.10, .41	.344	.02	−.26, .32	.875	−.02	−.31, .25	.863	−.01	−.28, .29	.924

*Note*. Correlations with *p* < .05 and bootstrapped BCa 95% CI not containing zero in bold. *r* Pearson correlation coefficient. *BCa 95% CI* Bootstrapped bias-corrected and accelerated 95% confidence interval based on 2000 bootstrap samples. *T0* pre-treatment. *LEC-5* Life Events Checklist for DSM-5 (weighted number of traumatic events). *BDI-II* Beck Depression Inventory – second edition. *BAI* Beck Anxiety Inventory. *PCL-5* PTSD Checklist for DSM-5.

### PTSD symptoms and hair ECs/NAEs across inpatient treatment

Table 3 shows hair ECs/NAEs and PTSD symptoms across treatment and follow-up. FDR-corrected post-hoc tests of the LMM adjusting for treatment duration, group (CBM-APP yes/no), and lifetime trauma exposure showed that PTSD symptoms declined significantly from pre- to post-treatment (*b* = −17.21, *p* < .001), pre-treatment to follow-up (*b* = −15.13, *p* < .001), but not from post-treatment to follow-up (*b* = 2.08, *p* = .443). Further, patients with more traumatic experiences had higher PTSD symptoms on average across time (*b* = 0.61, *p* = .028; see Table 3 for descriptive results and supplements Table S6 for full LMM results).

**Table 3:** PTSD symptoms and hair EC/NAE raw data (pg/mg) at pre-treatment, post-treatment, and follow-up.

	Pre-treatment (T0) (*M, SD, Mdn*, Range)	Post-treatment (T1) (*M, SD, Mdn*, Range)	Follow-up (T2) (*M, SD, Mdn*, Range)
PTSD symptoms (PCL-5)	(*n* = 54) 55.72 ± 8.88, 55.92 (35 – 69)	(*n* = 54) 38.23 ± 19.52, 39.50 (6 – 75)	(*n* = 40) 40.88 ± 19.85, 44.00 (1 – 75)
AEA	(*n* = 53) 0.74 ± 0.87, 0.46 (0.06 – 4.73)	(*n* = 53) 0.52 ± 0.44, 0.40 (0.06 – 1.85)	(*n* = 38) 0.69 ± 0.94, 0.39 (0.06 – 4.17)
1-AG/2-AG	(*n* = 53) 13.09 ± 8.46, 10.40 (1.93 – 42.55)	(*n* = 53) 13.34 ± 9.09, 10.79 (2.29 – 45.87)	(*n* = 38) 12.31 ± 5.97, 10.98 (2.89 – 30.74)
SEA	(*n* = 53) 4517.31 ± 3704.77, 2911.82 (937.31 – 15427.80)	(*n* = 53) 5346.00 ± 6079.29, 3131.60 (443.39 – 35564.40)	(*n* = 38) 4299.99 ± 5122.89, 2359.87 (684.21 – 24876.45)
PEA	(*n* = 53) 13505.05 ± 11904.95, 9161.64 (2841.75 – 52473.15)	(*n* = 53) 15286.28 ± 17413.86, 9606.87 (2109.92 – 103295.25)	(*n* = 38) 13539.25 ± 14590.40, 7536.11 (2014.34 – 67500.00)
OEA	(*n* = 53) 15974.02 ± 16309.17, 11404.40 (1674.68 – 79234.20)	(*n* = 53) 19132.52 ± 26803.13, 9434.88 (1320.66 – 162472.50)	(*n* = 38) 18845.05 ± 25852.50, 7918.97 (1376.46 – 102699.90)

Regarding the ECs/NAEs, average AEA levels trended toward being higher at pre-treatment than at post-treatment in FDR-corrected post-hoc tests (*b* = −0.10, *p* = .053), and SEA levels were higher pre-treatment (*b* = −0.12, *p* = .028) and post-treatment (*b* = −0.15, *p* = .013) compared to follow-up. 1-AG/2-AG, PEA, and OEA showed no significant differences between assessments (all *ps* > .05). However, regarding the NAEs, a significant effect of lifetime trauma exposure (SEA*: b* = 0.02, *p* = .009; PEA*: b* = 0.02, *p* = .003; OEA*: b* = 0.02, *p* = .002) and treatment duration (PEA*: b* = 0.01, *p* = .029) emerged, indicating more traumatic events and longer treatment duration (only PEA) to be associated with higher NAE levels (see Table 3 for descriptive results and supplements Table S6 for LMM results).

### Predicting PTSD symptoms in response to inpatient treatment

#### Pre-treatment hair ECs/NAEs

LMMs including the interaction of time with pre-treatment hair EC/NAE levels, controlling for group (CBM-APP yes/no), treatment duration, and lifetime trauma exposure showed that PTSD symptoms did not change significantly differently depending on the level of pre-treatment ECs/NAEs (all *ps* ≥ .449; see supplements Table S7).

#### Change in hair ECs/NAEs across treatment and follow-up

Results from LMMs adjusting for group (CBM-APP yes/no), treatment duration, and lifetime trauma exposure and including the interaction between time and the change in hair EC/NAE levels across treatment (*ps* ≥ .299) and follow-up (*ps* ≥ .081) indicated that PTSD symptoms did not change significantly differently depending on the change in hair EC/NAE levels (see supplements Table S8).

### Exploratory analyses in the subsample with exposure therapy

When repeating the analyses above only amongst patients who had received exposure treatment (*n* = 37), results remained the same (data not shown).

## Discussion

This secondary investigation on RCT data [68] explored hair ECs/NAEs in relation to lifetime trauma, clinical symptoms, and PTSD symptom change in female individuals with PTSD receiving inpatient treatment. Tentative pre-treatment associations emerged, with higher hair AEA levels associated with lower depressive and anxiety symptoms, but higher PTSD symptoms (after controlling for depressive symptoms), and higher SEA, PEA, and OEA levels associated with more lifetime trauma exposure. This distinction regarding the direction of associations is surprising and in part contrasts prior research and our hypothesis that a hypoactive ECS is linked to stress-related psychopathology [12, 23]. Additionally, longitudinal findings showed PTSD symptom improvement across treatment, remaining stable at follow-up. However, neither pre-treatment hair ECs/NAEs, nor their changes predicted treatment-induced PTSD symptom reduction. Overall, these findings extend prior research examining ECS alterations in PTSD, indicating that hair EC/NAE levels may not serve as an informative intervention-related biomarker for PTSD treatment response over 3 to 5 months in inpatient settings. Instead, results highlight that it may be necessary to combine markers from multiple biological systems relevant to PTSD to inform personalized treatment approaches in highly burdened samples.

### Pre-treatment associations

Overall, while some hair ECs/NAEs demonstrated isolated associations with trauma and clinical measures at pre-treatment, the findings should be interpreted cautiously due to the small sample size, wide confidence intervals, and inconsistent relationships across measures. In detail, our results tentatively indicated a relationship between higher hair NAE and AEA levels with greater trauma exposure and more severe PTSD-*distinct* symptoms (i.e., after accounting for the degree of concurrent depressive symptoms) respectively. This latter finding may be primarily influenced by hyperarousal symptoms, which are distinct from the overlapping features of depression and PTSD and showed a trend toward a positive association with hair AEA levels in exploratory subscale analyses. While the positive direction opposes our hypothesis and contrasts prior hair-based research showing a negative relationship between hair NAEs and lifetime trauma and PTSD symptom severity in Ugandan rebel war survivors [41], results may indicate that elevations in hair AEA represent an attempt to compensate high hyperarousal symptoms [46]. Our findings are consistent with previous hair-based research in chronically stressed, non-acutely trauma-exposed emergency medical personnel showing a positive association between the number of major life events and hair OEA and PEA [59]. Also, results are consistent with studies using short-term blood-based EC assessments that reported elevated ECs/NAEs in patients with both acute and chronic PTSD [45–47] and positive associations of PEA, 2-AG, and AEA with PTSD symptom severity, including specifically arousal symptoms [46, 48, 50]. Additionally, a recent meta-analysis identified higher baseline AEA and 2-AG in patients with stress-related neuropsychiatric disorders compared to controls [49] and our results partly align with findings of reduced blood EC levels only in male – but not female – patients with PTSD [52], suggesting possible sex-dependent biological alterations in PTSD [51].

Considering the comparatively high burden of our sample and the chronic nature of the PTSD symptoms as evident in an average PCL-5 score above 55 at pre-treatment and patients endorsing on average 7 lifetime traumatic events, our findings indicate that more cumulative trauma and greater PTSD-*distinct* symptom severity and the associated chronic stressful state may be associated with elevated long-term integrated hair AEA and NAE levels. Variation in ECS alterations in PTSD over time and with traumatic experiences may partly explain the heterogeneity in the literature. A recent study conducting metabolomics, proteomics, and DNA methylome assay in recent-onset and chronic PTSD cohorts identified distinct signaling and metabolic pathway activity, suggesting an initial compensatory response in the acute phase which changes with chronicity [94]. Also, a recent investigation demonstrated that individuals who developed high and severe comorbid PTSD and depressive symptoms had elevated plasma AEA levels shortly after trauma which were not reduced after 6 months [95]. A previous study has linked lower hair NAE levels greater lifetime trauma and PTSD symptoms in individuals with a high trauma load between 17 and 36 experienced traumatic events [41]. In contrast, the positive associations between hair AEA and PTSD-*distinct* symptoms and hair NAEs and trauma exposure in our chronic PTSD sample - with on average 7 traumatic events - suggest that the relationship between hair ECs/NAEs and traumatic stressors and PTSD-*distinct* symptoms may shift with cumulative trauma exposure. This may fit with the building block effect’s postulated long-term reduction in cortisol following trauma [39].

It should also be considered that EC/NAE levels were possibly elevated already prior to trauma exposure, for instance due to genetic polymorphisms of ECS-related enzymes and receptors [45, 48], which were not assessed in our study. An exciting future extension that combines different sources of information, such as genetic polymorphisms as well as relevant predictors, could involve the application of k-means clustering, a machine learning approach, to develop clinically predictive algorithms based in part on biological and trauma history data to better identify and support individuals at risk for more severe PTSD symptoms [96]. Finally, the lifetime trauma measure (i.e., LEC-5) did not differentiate traumatic events by whether they occurred during childhood, adolescence, or adulthood. However, childhood traumatic experiences have been linked to elevated hair 1-AG and reduced SEA in a healthy community sample of new mothers [97], while patients suffering from PTSD with childhood trauma had elevated hair OEA compared to healthy controls [47]. Given the threefold risk of PTSD in adulthood from childhood maltreatment [98], future research should assess age at trauma exposure, and trauma type, using for instance the Maltreatment and Abuse Chronology of Exposure Scale [99] or the Trauma History Questionnaire [100] when focusing on childhood or the lifespan respectively.

Cross-sectional findings also revealed that, consistent with our hypothesis, lower pre-treatment hair AEA was associated with higher levels of pre-treatment anxiety and depressive symptoms, particularly the affective and cognitive subscales of the BDI-II. This aligns with previous investigations demonstrating a negative relationship between ECs/NAEs and anxiety symptoms (e.g., hair SEA, PEA: [58]; blood 2-AG: [101]; hair AEA: [54]; blood AEA: [102]; blood AEA: [103]) and depressive symptoms (e.g., blood 2-AG: [102, 104]; hair AEA: [57]), yet contrasts recent meta-analytic results reporting elevated baseline AEA and 2-AG in individuals with anxiety, depression, and PTSD compared to healthy controls [49]. However, meta-analytic findings were highly heterogenous and recent systematic reviews underscore the importance of considering gender, symptom severity, chronicity and psychiatric comorbidities [105, 106]. Our findings align with a review of preclinical studies concluding that ECS activation and increased EC signaling induces antidepressant- and anxiolytic-like behaviors in animal models of depression [70]. This fits with the idea that EC downregulation may be related to increased risk for stress-related psychopathology [23], albeit clinical human evidence for pharmacological agents targeting the ECS in depression remaining limited to date [34].

### Treatment effects

Our results, like the primary RCT findings [68], showed a significant reduction in PTSD symptoms from pre-treatment to post-treatment, which remained stable to follow-up. This supports trauma-focused CBT-based treatment for PTSD as effective [5, 107]. However, average symptoms remained above cut-off at follow-up, highlighting the high burden of the sample. Our results suggest that individuals with greater lifetime trauma exposure have elevated PTSD symptoms on average, which aligns with research showing that a greater trauma load increases PTSD risk in a dose-dependent manner [37] and is associated with elevated PTSD symptoms across treatment, however not with a differential therapeutic response [108].

Given the important function attributed to the ECS, particularly AEA, in extinction learning [33, 63], we proposed that lower EC/NAE levels before trauma-focused therapy and EC/NAE decrease during and after treatment may be indicative of a diminished treatment effect. Contrary to this, pre-treatment hair ECs/NAEs levels and EC/NAE changes were not significantly associated with PTSD symptom changes across treatment and follow-up neither in the full sample nor in the subsample that had received exposure therapy. This aligns with a parallel investigation focusing on the same sample finding no evidence regarding stress-related hormones cortisol, cortisone, and DHEA measured in hair as an invention-related biomarker in this context [69] and one other study examining pre-treatment blood EC levels and treatment response, finding no significant relationship in war veterans [50]. Our lack of findings could be due to hair EC/NAE levels not being sufficiently sensitive to changes in such a short time. Thus, it may be promising for future research to combine short- and long-term assessments as well as assessments of EC/NAE responses under stressful conditions and during exposure therapy sessions [50]. This could improve our grasp of how exactly ECS functioning impacts specific PTSD treatment elements, thereby offering novel indicators that may inform a more personalized approach in PTSD treatment. Another avenue for precision and personalized medicine in PTSD treatment are omics studies, where high-throughput technology allows hypothesis-free examination of many biological markers, for instance genes, RNA transcripts, proteins, and metabolites [109]. Building on a metabolomics study in PTSD that implicated amongst others reduced PEA as having the strongest predictive association with PTSD diagnostic status [43], a next step could be to apply omics studies to PTSD treatment response to highlight relevant predictive biomolecular pathways.

### Strengths and limitations

To our knowledge this is the first study to investigate *hair* ECs/NAEs in a clinical sample of patients with PTSD in a naturalistic treatment setting representative of individuals with chronic PTSD in routine clinical care, enabling a considerable degree of external validity. Also, all patients received specialized trauma-focused treatment, and we included both post-treatment and 3-month follow-up assessments. Limitations include that the study was not originally designed to test the hypotheses, generalizability is reduced due to our all-female sample, and methodological restrictions remain. While analysis of scalp-near 3-cm hair segments, reflecting EC/NAE secretion three months prior to sampling, was well suited for pre-treatment and follow-up, it may have led to an overlap in the detection of EC/NAE levels between pre-treatment and post-treatment as the average gap was one month. This may render results relating to post-treatment to follow-up more meaningful and robust. Also, it remains uncertain to what extent hair ECs/NAEs reflect peripheral circulating levels [110]. As is common amongst psychosomatic inpatients [111], comorbid mental and physical health conditions, along with psychotropic medication use, were frequent in our sample and may have confounded results. We also did not assess patients’ diet and nutrient quality, which may impact EC/NAE profiles [112], or the use of exogenous cannabinoids, which is more likely among individuals with PTSD [113], and in turn, may downregulate the ECS [50]. Additionally, we did not consider menstrual health, such as premenstrual syndrome and its severity, which may be linked to altered ECS signalling via its modulation of mood, pain, and stress and interactions with steroid hormones, such as estrogen and progesterone [114]. Also, treatment protocols were not standardized (i.e., some did no exposure), likely due to symptom severity necessitating individual adaptations. While these methodological limitations hamper clear conclusions, the present study did investigate a sample representative of inpatients with PTSD for whom it would be the aim to better predict treatment response. Follow-up investigations including both a waitlist control group and a healthy control group with and without prior trauma would allow more detailed assessment of treatment effects and ECS alterations as diagnostic- and intervention-related markers. Also, external evaluation of symptom improvement by hospital staff, standardized treatment protocols, and larger samples enabling subgroup analyses would allow a more detailed exploration.

## Conclusion

Overall, cross-sectional findings tentatively confirmed previous research showing reduced hair AEA levels related to more depressive and anxiety symptoms. Regarding trauma-related measures, contrary to hypotheses, results indicated more PTSD-*distinct* symptoms and more lifetime trauma exposure associated with higher hair AEA and NAE levels respectively. These findings underscore the complexity of ECS alterations in stress-related psychopathology and their potential dependence on timing and severity, highlighting the need for further research. This study found no evidence that pre-treatment hair ECs/NAEs or their changes predict PTSD inpatient treatment response. However, the absence of a control group and the all-female sample limit generalizability. Overall, long-term integrated ECs/NAEs may not serve as key predictors of treatment response in female inpatients with PTSD. Given the limitations, larger-scale studies are needed to validate our findings.

## Supplementary information

Supplementary Materials

## Data Availability

Anonymized outcome data were based on the RCT and are available at: https://osf.io/jvstf/.
