# Supplementary material for: Endocannabinoid and N-acylethanolamine concentrations in hair of female patients with posttraumatic stress disorder – associations with clinical symptoms and outcomes following multimodal trauma-focused inpatient treatment
Source: Transl Psychiatry. 2025 Aug 23;15:312. doi: 10.1038/s41398-025-03476-3 (PMC12374999; doi:10.1038/s41398-025-03476-3)
Supplement: Supplementary file 1 — Supplementary Materials [file 41398_2025_3476_MOESM1_ESM.docx]

**Supplementary materials for the study titled:**

“**Endocannabinoid and *N*-acylethanolamine concentrations in hair of female patients with posttraumatic stress disorder – associations with clinical symptoms and outcomes following multimodal trauma-focused inpatient treatment**”

Table of Contents

[Table S1 2](#_Toc188961554)

[Table S2 3](#_Toc188961555)

[Table S3 4](#_Toc188961556)

[Table S4 5](#_Toc188961557)

[Table S5 6](#_Toc188961558)

[Table S6 7](#_Toc188961559)

[Table S7 9](#_Toc188961560)

[Table S8 11](#_Toc188961561)

[Information S1 14](#_Toc188961562)

# **Table S1**

*Results from attrition analyses for predictors using Welch test (N = 54).*

| Variable | *t* | *df* | *p* | Difference | 95% CI | *d* |
| --- | --- | --- | --- | --- | --- | --- |
|  | | | | | | |
| *Sociodemographic variables* | | | | | | |
| BMI | -0.96 | 22.89 | .35 | -3.01 | -9.52, 3.51 | 9.50 |
| Age | 0.98 | 21.58 | .34 | 3.14 | -3.55, 9.83 | 9.40 |
| Treatment Duration | -0.03 | 27.81 | .98 | -0.11 | -7.74, 7.52 | 12.40 |
|  |  |  |  |  |  |  |
| *Clinical variables* | | | | | | |
| BDI-II at T0 | -0.24 | 36.51 | .810 | -.55 | -5.18, 4.08 | 8.53 |
| BAI at T0 | -0.21 | 29.00 | .837 | -.67 | -7.31, 5.96 | 11.01 |
| PCL-5 at T0 | 0.27 | 25.02 | .788 | .77 | -5.06, 6.60 | 8.96 |
| PCL-5 at T1 | 1.17 | 28.21 | .250 | 6.81 | -5.07, 18.69 | 19.45 |
| LEC-5 | 0.05 | 29.77 | .961 | 0.11 | -4.24, 4.45 | 7.30 |
| CAPS-5 | 1.25 | 28.96 | .222 | 3.21 | -2.05, 8.47 | 8.73 |
|  |  |  |  |  |  |  |
| *Hair-related variables* | | | | | | |
| AEA at T0 | 1.19 | 32.01 | .243 | 0.14 | -0.10, 0.39 | 0.42 |
| 1-AG/2-AG at T0 | 0.99 | 33.47 | .328 | 0.08 | -0.08, 0.24 | 0.29 |
| SEA at T0 | **2.25** | **40.87** | **.030** | **0.20** | **0.02, 0.38** | **0.34** |
| PEA at T0 | 1.42 | 40.76 | .164 | 0.13 | -0.05, 0.31 | 0.35 |
| OEA at T0 | 0.84 | 42.37 | .404 | 0.09 | -0.13, 0.32 | 0.43 |
|  |  |  |  |  |  |  |
| AEA at T1 | 0.02 | 24.75 | .986 | 0.00 | -0.28, 0.28 | 0.42 |
| 1-AG/2-AG at T1 | 1.30 | 25.27 | .204 | 0.11 | -0.07, 0.29 | 0.27 |
| SEA at T1 | 0.89 | 26.92 | .380 | 0.11 | -0.14, 0.35 | 0.40 |
| PEA at T1 | 0.02 | 32.83 | .987 | 0.00 | -0.22, 0.22 | 0.38 |
| OEA at T1 | -0.05 | 34.04 | .959 | -0.01 | -0.28, 0.27 | 0.48 |
| *Note*. Two-tailed testing. Significant effects at *p* < .05 in bold. Completers (*n* = 38) and non-completers (*n =* 16) participated in the present RCT and provided hair samples, however only completers completed the follow-up assessment. Significant effects in bold. LEC-5 = Life Events Checklist for DSM-5 (weighted number of traumatic events). BDI-II = Beck Depression Inventory. BAI = Beck Anxiety Inventory. PCL-5 = PTSD Checklist for DSM-5. CAPS-5 = Clinician Administered PTSD Scale. T0 = pre-treatment. T1 = post-treatment. T2 = 3-month follow-up. | | | | | | |

# **Table S2**

*Results from attrition analyses for predictors using Pearson Chi-Square (N = 54).*

| Variable | Group | *n (%)* | $X^{2}$(df) | *p* | ϕ |
| --- | --- | --- | --- | --- | --- |
| Group (CBM-App) | Completers | 18 (47.4) | 0.36 (1) | .551 | .08 |
|  | No-completers | 9 (56.3) |  |  |  |
|  |  |  |  |  |  |
| Married | Completers | 13 (34.2) | 0.04 | .833 | -.03 |
|  | No-completers | 5 (31.3) |  |  |  |
|  |  |  |  |  |  |
| Number of F-diagnoses | Completers |  | 2.07 (1) | .150 | -.20 |
|  | 2 | 18 (47.4) |  |  |  |
|  | >3 | 20 (52.6) |  |  |  |
|  | No-completers |  |  |  |  |
|  | 2 | 11 (68.8) |  |  |  |
|  | 3 | 5 (31.3) |  |  |  |
|  |  |  |  |  |  |
| Migration background | Completers | 6 (15.8) |  | .537^a^ |  |
|  | No-completers | 3 (18.8) |  |  |  |
|  |  |  |  |  |  |
| Exposure therapy | Completers | 26 (68.4) | 0.00 (1) | .981 | .00 |
|  | Non-Completers | 11 (68.8) |  |  |  |
| *Note.* Two-sided significance testing. Completers (*n* = 38) and non-completers (*n =* 16) participated in the present RCT and provided hair samples, however only completers completed the follow-up assessment. *X^2^* = Pearson Chi-Square value. df = degrees of freedom. ϕ = Phi coefficient. ^a^ Fisher’s exact test because one expected cell count was less than 5. | | | | | |

# **Table S3**

*Pearson correlations between hair ECs/NAEs and potentially confounding variables*

| Hair EC/NAE |  | Hair mass | |  | Storage time | |  | BMI | |  | Age | |
| --- | --- | --- | --- | --- | --- | --- | --- | --- | --- | --- | --- | --- |
|  |  | *r* ^a^ | *p* |  | *r* | *p* |  | *r* | *p* |  | *r* | *p* |
| AEA T0 |  | -.08 | .569 |  | -.00 | .989 |  | -.23 | .103 |  | .09 | .542 |
| AEA T1 |  | .08 | .554 |  | -.12 | .381 |  | -.02 | .892 |  | .19 | .162 |
| AEA T2 |  | -.24 | .155 |  | .24 | .160 |  | .03 | .859 |  | .05 | .747 |
| 1-AG/2-AG T0 |  | -.05 | .718 |  | .03 | .842 |  | **-.34** | **.014** |  | .17 | .235 |
| 1-AG/2-AG T1 |  | .11 | .446 |  | -.14 | .322 |  | **-.30** | **.028** |  | .22 | .106 |
| 1-AG/2-AG T2 |  | -.29 | .079 |  | .10 | .543 |  | -.10 | .545 |  | .21 | .219 |
| SEA T0 |  | **-.29** | **.034** |  | -.00 | .996 |  | -.21 | .127 |  | .17 | .236 |
| SEA T1 |  | -.08 | .548 |  | -.01 | .931 |  | -.12 | .402 |  | .20 | .149 |
| SEA T2 |  | **-.37** | **.025** |  | .12 | .495 |  | .03 | .873 |  | .12 | .471 |
| PEA T0 |  | -.21 | .129 |  | .12 | .407 |  | -.11 | .430 |  | .20 | .150 |
| PEA T1 |  | -.20 | .153 |  | .03 | .858 |  | -.08 | .569 |  | .23 | .094 |
| PEA T2 |  | -.27 | .104 |  | .03 | .872 |  | .03 | .876 |  | .12 | .466 |
| OEA T0 |  | -.18 | .204 |  | .03 | .854 |  | -.15 | .297 |  | .22 | .112 |
| OEA T1 |  | -.18 | .190 |  | -.07 | .625 |  | -.09 | .534 |  | **.28** | **.043** |
| OEA T2 |  | -.32 | .056 |  | -.02 | .922 |  | .01 | .933 |  | .09 | .579 |
| *Note.* Significant correlations at *p* < .05 in bold. *^a^* point-biserial correlation. Hair mass coded as 0 ‘less than 7.5 mg’ and 1 ‘7.5 mg’. Two-tailed testing. Log-transformed AEA, 1AG/2AG, SEA, PEA, and OEA values were used. T0 = pre-treatment. T1 = post-treatment. T2 = 3-month follow-up. | | | | | | | | | | | | |

# **Table S4**

*Pearson correlations between hair EC/NAE*

|  | AEA T0 | AEA T1 | AEA T2 | 1AG/2AG T0 | 1AG/2AG T1 | 1AG/2AG T2 | SEA T0 | SEA T1 | SEA T2 | PEA T0 | PEA T1 | PEA T2 | OEA T0 | OEA T1 | OEA T2 |
| --- | --- | --- | --- | --- | --- | --- | --- | --- | --- | --- | --- | --- | --- | --- | --- |
| AEA T0 | - | **.60^***^** | **.51^***^** | **.54^***^** | **.40^**^** | **.50^**^** | .15 | .08 | -.20 | .09 | .04 | -.28 | .12 | .06 | -.28 |
| AEA T1 |  | - | **.63^***^** | **.42^**^** | **.48^***^** | **.54^***^** | -.09 | -.01 | -.13 | -.14 | .01 | -.13 | -.07 | .09 | -.13 |
| AEA T2 |  |  | - | **.39^*^** | **.34^*^** | **.55^***^** | .07 | .17 | **.34^*^** | .10 | .22 | .17 | .13 | .25 | .17 |
| 1-AG/2-AG T0 |  |  |  | - | **.75^***^** | **.60^**^** | **.33^*^** | .14 | .10 | .11 | -.05 | -.03 | .22 | .02 | .01 |
| 1-AG/2-AG T1 |  |  |  |  | - | **.73^***^** | .19 | .27 | .05 | -.11 | -.04 | -.03 | .00 | .08 | .06 |
| 1-AG/2-AG T2 |  |  |  |  |  | - | .13 | .00 | .16 | .01 | -.11 | -.04 | .04 | -.05 | .02 |
| SEA T0 |  |  |  |  |  |  | - | **.69^***^** | **.39^*^** | **.82^***^** | **.59^***^** | **.34^*^** | **.78^***^** | **.56^***^** | .29 |
| SEA T1 |  |  |  |  |  |  |  | - | **.56^***^** | **.55^***^** | **.80^***^** | **.49^**^** | **.50^***^** | **.72^***^** | **.45^**^** |
| SEA T2 |  |  |  |  |  |  |  |  | - | **.43^**^** | **.55^***^** | **.90^***^** | **.41^*^** | **.49^**^** | **.87^***^** |
| PEA T0 |  |  |  |  |  |  |  |  |  | - | **.72^***^** | **.42^**^** | **.95^***^** | **.67^***^** | **.36^*^** |
| PEA T1 |  |  |  |  |  |  |  |  |  |  | - | **.62^***^** | **.70^***^** | **.94^***^** | **.57^***^** |
| PEA T2 |  |  |  |  |  |  |  |  |  |  |  | - | **.43^**^** | **.58^***^** | **.97^***^** |
| OEA T0 |  |  |  |  |  |  |  |  |  |  |  |  | - | **.72^***^** | **.41^*^** |
| OEA T1 |  |  |  |  |  |  |  |  |  |  |  |  |  | - | **.57^***^** |
| OEA T2 |  |  |  |  |  |  |  |  |  |  |  |  |  |  | - |
| *Notes.* Significant correlations (*r*) in bold. ^*^ *p* < .05; ^**^ *p* < .01; ^***^ *p* < .001. Log-transformed hair EC/NAE values were used. T0 = pre-treatment. T1 = post-treatment. T2 = 3-month follow-up. | | | | | | | | | | | | | | | |

# **Table S5**

*Exploratory Pearson correlations between pre-treatment hair ECs/NAEs and BDI and PCL5 subscales (N = 53)*

|  |  | AEA T0 | | |  | AG T0 | | |  | SEA T0 | | |  | PEA T0 | | |  | OEA T0 | | |
| --- | --- | --- | --- | --- | --- | --- | --- | --- | --- | --- | --- | --- | --- | --- | --- | --- | --- | --- | --- | --- |
|  |  | *r* | BCa 95% CI | *p* |  | *r* | BCa 95% CI | *p* |  | *r* | BCa 95% CI | *p* |  | *r* | BCa 95% CI | *p* |  | *r* | BCa 95% CI | *p* |
| BDI-II affective |  | **-.32** | **-.53, -.11** | **.008** |  | -.06 | -.38, .29 | .724 |  | .04 | -.24, .31 | .809 |  | -.15 | -.39, .12 | .278 |  | -.12 | -.39, .18 | .394 |
| BDI-II cognitive |  | **-.26** | **-.46, -.06** | **.012** |  | -.25 | -.49, .04 | .077 |  | -.08 | -.31, .16 | .544 |  | -.15 | -.39, .09 | .230 |  | -.11 | -.35, .14 | .441 |
| BDI-II somatic |  | -.11 | -.33, .14 | .345 |  | .01 | -.28, .33 | .919 |  | .20 | -.09, .48 | .168 |  | .06 | -.26, .35 | .704 |  | .09 | -.24, .39 | .609 |
|  |  |  |  |  |  |  |  |  |  |  |  |  |  |  |  |  |  |  |  |  |
| PCL-5 intrusion |  | .07 | -.22, 34 | .639 |  | -.04 | -.30, .23 | .804 |  | -.08 | -.32, .16 | .545 |  | -.10 | -.34, .12 | .395 |  | -.09 | -.33, .13 | .464 |
| PCL-5 avoidance |  | .06 | -.20, .32 | .694 |  | .12 | -.17, .39 | .408 |  | .05 | -.24, .31 | .747 |  | -.06 | -.33, .20 | .675 |  | -.07 | -.35, .20 | .640 |
| PCL-5 mood and cognitions |  | -.04 | -.33, .25 | .738 |  | .01 | -.25, .27 | .934 |  | .04 | -.24, .32 | .768 |  | .00 | -.25, .25 | .999 |  | -.00 | -.26, .26 | .963 |
| PCL-5 hyperarousal |  | .24 | -.02, .48 | .074 |  | .10 | -.14, .33 | .415 |  | .07 | -.18, .33 | .548 |  | -.05 | -.32, .21 | .734 |  | .02 | -.23, .25 | .887 |
| *Note*. Correlations with *p* < .05 and bootstrapped BCa 95% CI not containing zero in bold. *r* = Pearson correlation coefficient. BCa 95% CI = Bootstrapped bias-corrected and accelerated 95% confidence interval based on 2000 bootstrap samples. T0 = pre-treatment. T1 = post-treatment. T2 = 3-month follow-up. BDI-II affective = affective symptom subscale of the Beck Depression Inventory – second edition. BDI-II cognitive = cognitive symptom subscale of the Beck Depression Inventory – second edition. BDI-II somatic = somatic symptom subscale Beck Depression Inventory – second edition. PCL-5 intrusion = intrusion subscale of the PTSD Checklist for DSM-5. PCL-5 avoidance = avoidance subscale of the PTSD Checklist for DSM-5. PCL-5 mood and cognition = changes in mood and cognition subscale of the PTSD Checklist for DSM-5. PCL-5 hyperarousal = hyperarousal subscale of the PTSD Checklist for DSM-5. | | | | | | | | | | | | | | | | | | | | |

# **Table S6**

*Results from robust linear mixed models predicting PTSD symptoms (PCL-5) and hair EC/NAE levels across time, controlling for treatment duration, group (CBM-APP yes/no), and lifetime trauma exposure, N =* 54

| **Outcome** | **Predictor** | *b* | 95% CI (*b*) | β | 95% CI (β) | t | *p* |
| --- | --- | --- | --- | --- | --- | --- | --- |
| PCL-5 | (Intercept) | **57.56** | **51.45, 63.66** | **.67** | **.34, 1.00** | **18.64** | **<.001** |
|  | Time (T0 vs. T1) | **-17.21** | **-22.05, -12.37** | **-.94** | **-1.21, -.68** | **-7.03** | **<.001** |
|  | Time (T0 vs. T2)^1^ | **-15.13** | **-20.50, -9.75** | **-.83** | **-1.12, -.53** | **-5.56** | **<.001** |
|  | Group | -4.33 | -12.08, 3.41 | -.24 | -.66, .19 | -1.11 | .271 |
|  | Treatment Duration | -0.01 | -0.43, 0.41 | -.01 | -.22, .20 | -0.06 | .952 |
|  | Lifetime Trauma Exposure | **0.61** | **0.07, 1.15** | **.24** | **.03, .46** | **2.22** | **.028** |
| Marginal $R^{2}$ = .23, Conditional $R^{2}$ = .59 | | | | | | | |
| *AEA* | (Intercept) | **-0.37** | **-0.52, -0.23** | **.03** | **-.31, .38** | **-5.12** | **<.001** |
|  | Time (T0 vs. T1) | **-0.10** | **-0.18, -0.02** | **-.24** | **-.43, -.04** | **-2.37** | **.019** |
|  | Time (T0 vs. T2)^2^ | -0.08 | -0.17, .0.02 | -.18 | -.41, .04 | -1.62 | .107 |
|  | Group | 0.07 | -0.12, 0.26 | .17 | -.29, .63 | 0.74 | .460 |
|  | Treatment Duration | -0.00 | -0.01, 0.01 | -.10 | -.32, .13 | -0.86 | .394 |
|  | Lifetime Trauma Exposure | 0.00 | -0.01, 0.02 | .07 | -.15, .30 | 0.64 | .526 |
| Marginal $R^{2}$ = .04, Conditional $R^{2}$ = .72 | | | | | | | |
| 1-AG/2-AG | (Intercept) | **1.05** | **0.95, 1.14** | **.03** | **-.32, .39** | **21.88** | **<.001** |
|  | Time (T0 vs. T1) | -0.01 | -0.05, 0.04 | -.03 | -.20, -.14 | -0.31 | .758 |
|  | Time (T0 vs. T2)^3^ | -0.04 | -0.09, .0.01 | -.16 | -.36, .03 | -1.65 | .102 |
|  | Group | 0.02 | -0.11, 0.14 | .06 | -.42, .54 | 0.24 | .810 |
|  | Treatment Duration | -0.00 | -0.01, 0.01 | -.05 | -.29, .18 | -0.46 | .644 |
|  | Lifetime Trauma Exposure | 0.00 | -0.01, 0.01 | .10 | -.14, .34 | 0.82 | .411 |
| Marginal $R^{2}$ = .02, Conditional $R^{2}$ = .79 | | | | | | | |
| SEA | (Intercept) | **3.52** | **3.40, 3.65** | **.03** | **-.31, .37** | **56.33** | **<.001** |
|  | Time (T0 vs. T1) | 0.02 | -0.06, 0.11 | .07 | -.18, .31 | 0.55 | .583 |
|  | Time (T0 vs. T2)^4^ | **-0.12** | **-0.22, -0.02** | **-.33** | **-.61, -.05** | **-2.36** | **.020** |
|  | Group | -0.03 | -0.19, 0.13 | -.09 | -.52, .35 | -0.41 | .684 |
|  | Treatment Duration | 0.01 | -0.00, 0.02 | .20 | -.01, .41 | 1.88 | .062 |
|  | Lifetime Trauma Exposure | **0.02** | **-0.00, 0.03** | **.29** | **.07, .50** | **2.64** | **.009** |
| Marginal $R^{2}$ = .14, Conditional $R^{2}$ = .61 | | | | | | | |
| PEA | (Intercept) | **3.94** | **3.82, 4.07** | **-.11** | **-.45, .23** | **62.31** | **<.001** |
|  | Time (T0 vs. T1) | 0.01 | -0.07, 0.10 | .04 | -.19, .27 | 0.33 | .744 |
|  | Time (T0 vs. T2)^5^ | -0.08 | -0.17, 0.02 | -.21 | -.47, .05 | -1.57 | .118 |
|  | Group | 0.08 | -0.09, 0.24 | .21 | -.24, .65 | 0.92 | .357 |
|  | Treatment Duration | **0.01** | **0.00, 0.02** | **.24** | **.03, .45** | **2.21** | **.029** |
|  | Lifetime Trauma Exposure | **0.02** | **0.01, 0.03** | **.33** | **.11, .55** | **2.98** | **.003** |
| Marginal $R^{2}$ = .18, Conditional $R^{2}$ = .66 | | | | | | | |
| OEA | (Intercept) | **3.93** | **3.77, 4.09** | **-.15** | **-.50, .19** | **48.88** | **<.001** |
|  | Time (T0 vs. T1) | 0.01 | -0.10, 0.12 | .02 | -.22, .26 | 0.19 | .850 |
|  | Time (T0 vs. T2)^6^ | -0.06 | -0.19, 0.06 | -.13 | -.40, .14 | -0.96 | .340 |
|  | Group | 0.12 | -0.09, 0.32 | .26 | -.19, .70 | 1.13 | .261 |
|  | Treatment Duration | 0.01 | -0.00, 0.02 | .16 | -.06, .38 | 1.47 | .144 |
|  | Lifetime Trauma Exposure | **0.02** | **0.01, 0.04** | **.35** | **.13, .57** | **3.11** | **.002** |
| Marginal $R^{2}$ = .16, Conditional $R^{2}$ = .65 | | | | | | | |
| *Note*. Significant effects with *p* < .05 in bold. Continuous variables were mean-centred to improve interpretation. T0 = pre-treatment. T1 = post-treatment. T2 = 3-month follow-up. ^1^ PCL-5: Time (T1 vs. T2): *b* = 2.08, *SE* = 2.72, *p* = .443; ^2^ AEA: Time (T1 vs. T2): *b* = 0.02, *SE* = 0.05, *p* = .647; ^3^ 1-AG/2-AG: Time (T1 vs. T2): *b* = -0.04, *SE* = 0.03, *p* = .252; ^4^ **SEA:** **Time (T1 vs. T2): *b* = -0.15, *SE* = 0.05, *p* = .013**; ^5^ PEA: Time (T1 vs. T2): *b* = 0.09, *SE* = 0.05, *p* = .174; ^6^ OEA: Time (T1 vs. T2): *b* = 0.07, *SE* = 0.06, *p* = .507. Marginal $R^{2}$ = proportion of variance explained by the fixed effects. Conditional $R^{2}$ = proportion of variance explained by both the fixed and random effects. | | | | | | | |

# **Table S7**

*Results from robust linear mixed models predicting PTSD symptoms (PCL-5) across time from pre-treatment (T0) EC/NAE levels, N = 53*

| **EC/NAE** | **Predictor** | *b* | 95% CI (*b*) | β | 95% CI (β) | t | *p* |
| --- | --- | --- | --- | --- | --- | --- | --- |
| AEA | (Intercept) | **58.21** | **51.87, 64.56** | **.69** | **0.35, 1.03** | **18.14** | **<.001** |
|  | Time (T0 vs. T1) | **-17.70** | **-22.65, -12.74** | **-.96** | **-1.23, -0.69** | **-7.06** | **<.001** |
|  | Time (T0 vs. T2) | **-15.57** | **-21.09, -10.05** | **-.85** | **-1.14, -0.55** | **-5.58** | **<.001** |
|  | T0 AEA | 1.24 | -10.33, 12.82 | .03 | -0.24, 0.30 | 0.21 | .832 |
|  | Group | -4.70 | -12.70, 3.29 | -.26 | -0.69, 0.18 | -1.16 | .247 |
|  | Treatment Duration | -0.05 | -0.50, 0.40 | -.02 | -0.23, 0.19 | -0.22 | .829 |
|  | Lifetime Trauma Exposure | 0.66 | 0.07, 1.24 | .25 | 0.03, 0.47 | 2.22 | **.028** |
|  | Time (T0 vs. T1)^*^T0 AEA | -1.52 | -13.27, 10.23 | -.04 | -0.31, 0.24 | -0.26 | .798 |
|  | Time (T0 vs. T2)^*^T0 AEA | -3.94 | -16.51, 8.64 | -.09 | -0.38, 0.20 | -0.62 | .537 |
| Marginal $R^{2}$ = .23, Conditional $R^{2}$ = .60 | | | | | | | |
| 1-AG/2-AG | (Intercept) | **58.58** | **52.32, 64.83** | **.71** | **0.37, 1.05** | **18.51** | **<.001** |
|  | Time (T0 vs. T1) | **-17.62** | **-22.50, -12.74** | **-.96** | **-1.22, -0.69** | **-7.14** | **<.001** |
|  | Time (T0 vs. T2) | **-15.60** | **-21.03, -10.16** | **-.85** | **-1.14, -0.55** | **-5.68** | **<.001** |
|  | T0 1-AG/2-AG | 2.51 | -14.37, 19.40 | .04 | -0.23, 0.31 | 0.29 | .769 |
|  | Group | -5.38 | -13.34, 2.58 | -.29 | -0.72, 0.14 | -1.34 | .184 |
|  | Treatment Duration | -0.04 | -0.48, 0.40 | -.02 | -0.23, 0.19 | -0.18 | .856 |
|  | Lifetime Trauma Exposure | **0.67** | **0.10, 1.23** | **.25** | **0.04, 0.46** | **2.33** | **.021** |
|  | Time (T0 vs. T1)^*^T0 1-AG/2-AG | 4.68 | -12.43, 21.78 | .07 | -0.20, 0.34 | 0.54 | .590 |
|  | Time (T0 vs. T2)^*^T0 1-AG/2-AG | 3.73 | -14.38, 21.83 | .06 | -0.23, 0.35 | 0.41 | .685 |
| Marginal $R^{2}$ = .24, Conditional $R^{2}$ = .60 | | | | | | | |
| SEA | (Intercept) | **58.06** | **51.73, 64.39** | **.68** | **0.34, 1.02** | **18.13** | **<.001** |
|  | Time (T0 vs. T1) | **-17.63** | **-22.55, -12.71** | **-.96** | **-1.22, -0.69** | **-7.09** | **<.001** |
|  | Time (T0 vs. T2) | **-15.55** | **-21.06, -10.03** | **-.84** | **-1.14, -0.55** | **-5.57** | **<.001** |
|  | T0 SEA | -2.67 | -17.42, 12.08 | -.05 | -0.33, 0.23 | -0.36 | .721 |
|  | Group | -4.48 | -12.49, 3.52 | -.24 | -0.68, 0.19 | -1.11 | .270 |
|  | Treatment Duration | -0.00 | -0.47, 0.46 | -.00 | -0.22, 0.22 | -0.01 | .989 |
|  | Lifetime Trauma Exposure | **0.71** | **0.11, 1.32** | **.27** | **0.04, 0.49** | **2.34** | **.021** |
|  | Time (T0 vs. T1)^*^T0 SEA | -2.40 | -16.57, 11.76 | -.05 | -0.32, 0.23 | -0.34 | .738 |
|  | Time (T0 vs. T2)^*^T0 SEA | -1.48 | -16.77, 13.82 | -.03 | -0.32, 0.26 | -0.19 | .849 |
| Marginal $R^{2}$ = .24, Conditional $R^{2}$ = .60 | | | | | | | |
| PEA | (Intercept) | **58.07** | **51.76, 64.37** | **.68** | **0.34, 1.02** | **18.23** | **<.001** |
|  | Time (T0 vs. T1) | **-17.60** | **-22.48, -12.72** | **-.96** | **-1.22, -0.69** | **-7.13** | **<.001** |
|  | Time (T0 vs. T2) | **-15.92** | **-21.38, -10.45** | **-.86** | **-1.16, -0.56** | **-5.76** | **<.001** |
|  | T0 PEA | -5.21 | -20.05, 9.63 | -.10 | -0.39, 0.19 | -0.69 | .489 |
|  | Group | -4.37 | -12.38, 3.64 | -.24 | -0.67, 0.20 | -1.08 | .282 |
|  | Treatment Duration | 0.01 | -0.46, 0.48 | .00 | -0.22, 0.23 | 0.03 | .972 |
|  | Lifetime Trauma Exposure | **0.73** | **0.13, 1.33** | **.27** | **0.05, 0.50** | **2.40** | **.018** |
|  | Time (T0 vs. T1)^*^T0 PEA | -1.29 | -15.29, 12.71 | -.02 | -0.30, 0.25 | -0.18 | .856 |
|  | Time (T0 vs. T2)^*^T0 PEA | 4.85 | -10.15, 19.84 | .09 | -0.20, 0.38 | 0.64 | .524 |
| Marginal $R^{2}$ = .24, Conditional $R^{2}$ = .60 | | | | | | | |
| OEA | (Intercept) | **58.12** | **51.76, 64.48** | **.68** | **0.34, 1.03** | **18.08** | **<.001** |
|  | Time (T0 vs. T1) | **-17.60** | **-22.48, -12.72** | **-.95** | **-1.22, -0.69** | **-7.13** | **<.001** |
|  | Time (T0 vs. T2) | **-15.89** | **-21.34, -10.44** | **-.86** | **-1.15, -0.56** | **-5.76** | **<.001** |
|  | T0 OEA | -3.22 | -15.01, 8.58 | -.08 | -0.36, 0.21 | -0.54 | .591 |
|  | Group | -4.53 | -12.65, 3.60 | -.25 | -0.69, 0.19 | -1.10 | .272 |
|  | Treatment Duration | -0.03 | -0.49, 0.43 | -.01 | -0.23, 0.20 | -0.12 | .902 |
|  | Lifetime Trauma Exposure | **0.70** | **0.09, 1.30** | **.26** | **0.03, 0.49** | **2.28** | **.024** |
|  | Time (T0 vs. T1)^*^T0 OEA | 0.17 | -11.10, 11.43 | .00 | -0.27, 0.28 | 0.03 | .977 |
|  | Time (T0 vs. T2)^*^T0 OEA | 4.55 | -7.31, 16.41 | .11 | -0.18, 0.40 | 0.76 | .449 |
| Marginal $R^{2}$ = .24, Conditional $R^{2}$ = .61 | | | | | | | |
| *Note*. Significant effects *p* < .05 in bold. Continuous variables were mean-centred to improve interpretation. T0 = pre-treatment. T1 = post-treatment. T2 = 3-month follow-up. Marginal $R^{2}$ = proportion of variance explained by the fixed effects. Conditional $R^{2}$ = proportion of variance explained by both the fixed and random effects. Interaction effect Time (T1 vs. T2)*T0 EC/NAE all *p* > .420. | | | | | | | |

# **Table S8**

*Results from robust linear mixed models predicting PTSD symptoms across time from change in EC/NAE levels across (T0-T1; N = 53) and following treatment (T1-T2; N = 37)*

|  |  | T0-T1^a^ | | | | | |  | T1-T2^b^ | | | | | |
| --- | --- | --- | --- | --- | --- | --- | --- | --- | --- | --- | --- | --- | --- | --- |
| **EC/NAE** | **Predictor** | *b* | 95%  CI (*b*) | β | 95%  CI (β) | *t* | *p* |  | *b* | 95%  CI (*b*) | β | 95%  CI (β) | *t* | *p* |
| AEA | (Intercept) | **56.77** | **49.66, 63.87** | **.61** | **0.22, 0.99** | **15.79** | **<.001** |  | **58.00** | **45.06, 70.94** | **.67** | **-0.02, 1.36** | **8.89** | **<.001** |
|  | Time (T0 vs. T1) | **-16.21** | **-22.24, -10.19** | **-.88** | **-1.21, -0.55** | **-5.32** | **<.001** |  | **-17.18** | **-27.51, -6.84** | **-.92** | **-1.48, -0.37** | **-3.30** | **.001** |
|  | Time (T0 vs. T2) | **-16.59** | **-23.31, -9.88** | **-.90** | **-1.26, -0.54** | **-4.89** | **<.001** |  | **-11.14** | **-21.47, -0.80** | **-.60** | **-1.16, -0.04** | **-2.14** | **.035** |
|  | AEA Change | 4.92 | -5.24, 15.09 | .27 | -0.28, 0.82 | 0.96 | .340 |  | -0.31 | -13.85, 13.23 | -.02 | -0.75, 0.71 | -0.05 | .964 |
|  | Group | -5.39 | -13.45, 2.68 | -.29 | -0.73, 0.15 | -1.32 | .189 |  | -4.02 | -14.72, 6.68 | -.22 | -0.79, 0.36 | -0.75 | .457 |
|  | Treatment Duration | -0.08 | -0.54, 0.37 | -.04 | -0.25, 0.17 | -0.37 | .713 |  | 0.08 | -0.60, 0.77 | .03 | -0.25, 0.32 | 0.24 | .807 |
|  | Lifetime Trauma Exposure | **0.64** | **0.06, 1.22** | **.24** | **0.02, 0.46** | **2.18** | **.031** |  | 0.60 | -0.18, 1.38 | .22 | -0.07, 0.51 | 1.52 | .132 |
|  | Time (T0 vs. T1)^*^AEA Change | -4.21 | -14.27, 5.85 | -.23 | -0.77, 0.32 | -0.83 | .409 |  | 1.78 | -10.79, 14.35 | .10 | -0.58, 0.77 | 0.28 | .780 |
|  | Time (T0 vs. T2)^*^AEA Change | 3.20 | -8.00, 14.41 | .17 | -0.43, 0.78 | 0.57 | .573 |  | -6.19 | -18.77, 6.38 | -.33 | -1.01, 0.34 | -0.98 | .331 |
|  | Marginal $R^{2}$ = .25, Conditional $R^{2}$ = .62 | | | | | | |  | Marginal $R^{2}$ = .18, Conditional $R^{2}$ = .63 | | | | | |
| 1-AG/2-AG | (Intercept) | **57.81** | **48.99, 66.63** | **.67** | **0.19, 1.14** | **12.96** | **<.001** |  | **57.21** | **47.75, 66.67** | **.63** | **0.12, 1.14** | **12.00** | **<.001** |
|  | Time (T0 vs. T1) | **-17.44** | **-24.20, -10.67** | **-.95** | **-1.31, -0.58** | **-5.10** | **<.001** |  | **-13.62** | **-21.48, -5.76** | **-.73** | **-1.16, -0.31** | **-3.44** | **.001** |
|  | Time (T0 vs. T2) | **-14.70** | **-22.31, -7.08** | **-.80** | **-1.21, -0.38** | **-3.82** | **<.001** |  | **-14.13** | **-22.00, -6.27** | **-.76** | **-1.18, -0.34** | **-3.57** | **.001** |
|  | 1-AG/2-AG Change | 0.74 | -9.37, 10.86 | .04 | -0.51, 0.59 | 0.15 | .885 |  | 1.39 | -11.47, 14.25 | .07 | -0.62, 0.77 | 0.21 | .831 |
|  | Group | -4.65 | -13.18, 3.87 | -.25 | -0.71, 0.21 | -1.08 | .283 |  | -3.83 | -14.31, 6.66 | -.21 | -0.77, 0.36 | -0.72 | .471 |
|  | Treatment Duration | -0.05 | -0.49, 0.39 | -.02 | -0.23, 0.19 | -0.22 | .823 |  | 0.13 | -0.56, 0.82 | .05 | -0.23, 0.34 | 0.37 | .711 |
|  | Lifetime Trauma Exposure | **0.66** | **0.08, 1.23** | **.25** | **0.03, 0.46** | **2.25** | **.026** |  | 0.59 | -0.17, 1.35 | .22 | -0.06, 0.50 | 1.53 | .129 |
|  | Time (T0 vs. T1)^*^ 1-AG/2-AG Change | -0.48 | -10.33, 9.37 | -.03 | -0.56, 0.51 | -0.10 | .923 |  | -6.43 | -18.78, 5.92 | -.35 | -1.01, 0.32 | -1.03 | .304 |
|  | Time (T0 vs. T2)^*^  1-AG/2-AG Change | -1.84 | -12.79, 9.12 | -.10 | -0.69, 0.49 | -0.33 | .741 |  | -3.78 | -16.13, 8.57 | -.20 | -0.87, 0.46 | -0.61 | .545 |
|  | Marginal $R^{2}$ = .23, Conditional $R^{2}$ = .60 | | | | | | |  | Marginal $R^{2}$ = .18, Conditional $R^{2}$ = .61 | | | | | |
| SEA | (Intercept) | **57.37** | **48.26, 66.48** | **.64** | **0.15, 1.14** | **12.45** | **<.001** |  | **57.91** | **49.06, 66.75** | **.67** | **0.20, 1.14** | **12.99** | **<.001** |
|  | Time (T0 vs. T1) | **-19.99** | **-27.22, -12.77** | **-1.08** | **-1.48, -0.69** | **-5.47** | **<.001** |  | **-17.88** | **-24.87, -10.89** | **-.96** | **-1.34, -0.59** | **-5.07** | **<.001** |
|  | Time (T0 vs. T2) | **-18.63** | **-26.44, -10.83** | **-1.01** | **-1.43, -0.59** | **-4.72** | **<.001** |  | **-18.09** | **-25.08, -11.10** | **-.97** | **-1.35, -0.60** | **-5.14** | **<.001** |
|  | SEA Change | 0.43 | -9.61, 10.47 | .02 | -0.52, 0.57 | 0.09 | .932 |  | 0.92 | -13.04, 14.88 | .05 | -0.70, 0.80 | 0.13 | .896 |
|  | Group | -3.54 | -11.96, 4.89 | -.19 | -0.65, 0.26 | -0.83 | .408 |  | -4.82 | -15.72, 6.08 | -.26 | -0.85, 0.33 | -0.88 | .382 |
|  | Treatment Duration | -0.03 | -0.47, 0.42 | -.01 | -0.22, 0.20 | -0.12 | .903 |  | 0.13 | -0.56, 0.82 | .05 | -0.23, 0.34 | 0.38 | .706 |
|  | Lifetime Trauma Exposure | **0.64** | **0.07, 1.22** | **.24** | **0.03, 0.46** | **2.21** | **.029** |  | 0.62 | -0.16, 1.40 | .23 | -0.06, 0.52 | 1.58 | .117 |
|  | Time (T0 vs. T1)^*^SEA Change | 4.33 | -5.61, 14.26 | .23 | -0.30, 0.77 | 0.86 | .391 |  | 7.08 | -5.74, 19.89 | .38 | -0.31, 1.07 | 1.10 | .276 |
|  | Time (T0 vs. T2)^*^SEA Change | 5.83 | -5.22, 16.88 | .32 | -0.28, 0.92 | 1.04 | .299 |  | 8.59 | -4.22, 21.41 | .46 | -0.23, 1.15 | 1.33 | .187 |
|  | Marginal $R^{2}$ = .25, Conditional $R^{2}$ = .60 | | | | | | |  | Marginal $R^{2}$ = .19, Conditional $R^{2}$ = .64 | | | | | |
| PEA | (Intercept) | **56.51** | **48.47, 64.56** | **.60** | **0.17, 1.03** | **13.90** | **<.001** |  | **56.84** | **47.87, 65.81** | **.60** | **0.12, 1.08** | **12.57** | **<.001** |
|  | Time (T0 vs. T1) | **-16.28** | **-23.03, -9.54** | **-.88** | **-1.25, -0.52** | **-4.78** | **<.001** |  | **-18.27** | **-25.53, -11.01** | **-.98** | **-1.37, -0.59** | **-4.99** | **<.001** |
|  | Time (T0 vs. T2) | **-16.49** | **-23.70, -9.28** | **-.89** | **-1.29, -0.50** | **-4.52** | **<.001** |  | **-18.56** | **-25.82, -11.30** | **-1.00** | **-1.39, -0.61** | **-5.07** | **<.001** |
|  | PEA Change | 3.17 | -6.69, 13.03 | .17 | -0.36, 0.71 | 0.64 | .526 |  | 3.79 | -9.16, 16.74 | .20 | -0.49, 0.90 | 0.58 | .563 |
|  | Group | -4.46 | -12.42, 3.51 | -.24 | -0.67, 0.19 | -1.11 | .270 |  | -4.33 | -14.72, 6.06 | -.23 | -0.79, 0.33 | -0.83 | .411 |
|  | Treatment Duration | -0.01 | -0.47, 0.45 | -.00 | -0.22, 0.21 | -0.04 | .970 |  | 0.03 | -0.65, 0.70 | .01 | -0.27, 0.29 | 0.08 | .936 |
|  | Lifetime Trauma Exposure | **0.64** | **0.07, 1.22** | **.24** | **0.03, 0.46** | **2.21** | **.029** |  | 0.66 | -0.10, 1.41 | .24 | -0.04, 0.53 | 1.72 | .089 |
|  | Time (T0 vs. T1)^*^PEA Change | -2.99 | -12.81, 6.82 | -.16 | -0.69, 0.37 | -0.60 | .548 |  | 7.12 | -5.13, 19.36 | .38 | -0.28, 1.04 | 1.15 | .252 |
|  | Time (T0 vs. T2)^*^PEA Change | 2.38 | -8.66, 13.43 | .13 | -0.47, 0.73 | 0.43 | .670 |  | 8.50 | -3.75, 20.74 | .46 | -0.20, 1.12 | 1.38 | .172 |
|  | Marginal $R^{2}$ = .24, Conditional $R^{2}$ = .60 | | | | | | |  | Marginal $R^{2}$ = .22, Conditional $R^{2}$ = .64 | | | | | |
| OEA | (Intercept) | **56.12** | **47.93, 64.32** | **.58** | **0.13, 1.02** | **13.54** | **<.001** |  | **57.30** | **48.30, 66.30** | **.63** | **0.15, 1.11** | **12.63** | **<.001** |
|  | Time (T0 vs. T1) | **-16.13** | **-23.16, -9.11** | **-.87** | **-1.26, -0.49** | **-4.54** | **<.001** |  | **-18.87** | **-26.52, -11.22** | **-1.02** | **-1.43, -0.60** | **-4.89** | **<.001** |
|  | Time (T0 vs. T2) | **-17.57** | **-25.27, -9.88** | **-.95** | **-1.37, -0.54** | **-4.52** | **<.001** |  | **-19.95** | **-27.59, -12.30** | **-1.07** | **-1.49, -0.66** | **-5.17** | **<.001** |
|  | OEA Change | 3.75 | -5.95, 13.45 | .20 | -0.32, 0.73 | 0.76 | .446 |  | 3.41 | -9.16, 15.98 | .18 | -0.49, 0.86 | 0.54 | .591 |
|  | Group | -4.41 | -12.41, 3.59 | -.24 | -0.67, 0.19 | -1.09 | .278 |  | -5.43 | -15.86, 4.99 | -.29 | -0.85, 0.27 | -1.03 | .304 |
|  | Treatment Duration | -0.03 | -0.48, 0.42 | -.01 | -0.23, 0.20 | -0.13 | .893 |  | 0.06 | -0.60, 0.73 | .03 | -0.25, 0.30 | 0.19 | .850 |
|  | Lifetime Trauma Exposure | **0.64** | **0.06, 1.22** | **.24** | **0.02, 0.46** | **2.19** | **.030** |  | 0.63 | -0.12, 1.37 | .23 | -0.04, 0.51 | 1.68 | .097 |
|  | Time (T0 vs. T1)^*^OEA Change | -3.12 | -12.96, 6.72 | -.17 | -0.70, 0.36 | -0.63 | .531 |  | 7.00 | -5.01, 19.01 | .38 | -0.27, 1.02 | 1.16 | .250 |
|  | Time (T0 vs. T2)^*^OEA Change | 4.11 | -6.84, 15.06 | .22 | -0.37, 0.82 | 0.74 | .459 |  | 10.68 | -1.33, 22.69 | .57 | -0.07, 1.22 | 1.76 | .081 |
|  | Marginal $R^{2}$ = .24, Conditional $R^{2}$ = .61 | | | | | | |  | Marginal $R^{2}$ = .23, Conditional $R^{2}$ = .64 | | | | | |
| *Note*. Significant effects *p* < .05 in bold. Continuous variables were mean-centred to improve interpretation. ^a^ *N* = 53; ^b^ *N* = 37; T0 = pre-treatment. T1 = post-treatment. T2 = 3-month follow-up. Marginal $R^{2}$ = proportion of variance explained by the fixed effects. Conditional $R^{2}$ = proportion of variance explained by both the fixed and random effects. Interaction effect Time (T1 vs. T2)* EC/NAE Change all *p* > .193. | | | | | | | | | | | | | | |

# **Information S1**

*Detailed description of the hair EC/NAE analysis protocol*

First, hair segments were cut at 3 cm, then placed in Falcon tubes and washed by shaking them twice in 2.5 mL isopropanol for 3 minutes, at room temperature. Thereafter, tubes were placed below a fume hood for a minimum of 12 hours and then 7.5 mg (range 3.7 – 7.5mg) were weighed out and sliced up. For endocannabinoid extraction, 20 μL of internal standard and 1.8 mL methanol were added, and the sample was incubated for 18 hours at room temperature. Then a 1.6 mL aliquot of clear supernatant was transferred to a new tube and evaporated at 50 °C under a steady nitrogen stream for about 20 minutes until the samples were fully dried. The dried residue was then suspended with 120 μL of a 50/50 methanol/water solution (v/v), and 100 μL of this mixture was injected for liquid chromatography tandem mass spectrometry (LC-MS/MS) analysis.

The LC–MS/MS system was conducted on a Shimadzu ultra-high performance liquid chromatography (Shimadzu, Canby, OR, USA) and AB Sciex QTRAP 6500 mass spectrometer coupled with Electrospray Ionization (ESI) source in positive ionization mode (AB Sciex, Foster City, CA, USA). The LC gradient used methanol as mobile phase A, water containing 2.0 mM ammonium acetate as mobile phase B, water for mobile phase C-A, and methanol for mobile phase C-B, with the following gradient conditions: “20% eluent B for 4.5 min; 20-0% B from 4.5 to 6.7 min; 0% B from 6.7 to 8.5 min and then re-equilibrated at 20% B until 9.0 min.” ((1), p. 2). A 100 μL injection volume was used, and the column temperature was maintained at 40 °C. Quality control samples were included for each 96 well plate during sample analysis. During the method development, the inter- and intra-day coefficients of variation (CVs) for quality control samples were examined and were below the recommended cut-off of 15% (1).

1. Gao W, Walther A, Wekenborg M, Penz M, Kirschbaum C. Determination of endocannabinoids and N-acylethanolamines in human hair with LC-MS/MS and their relation to symptoms of depression, burnout, and anxiety. Talanta. 2020 Sep;217:121006.
